# Editorial on writing reviews for the *British Journal of Nutrition*

**DOI:** 10.1017/S0007114520000185

**Published:** 2020-05-14

**Authors:** Barbara A. Fielding, Jean-Paul Lalles, Gerald E. Lobley, Stefan M. Pasiakos, Susan J. Whiting

**Affiliations:** 1 Faculty of Health and Medical Sciences, University of Surrey, Guildford GU2 7WG, UK; 2 Human Nutrition Division, Institut National de la Recherche Agronomique (INRA), Clermont-Ferrand, France; 3 The Centre de Recherche en Nutrition Humaine Ouest, Nantes, France; 4 The Rowett Institute, University of Aberdeen, Foresterhill, Aberdeen AB25 2ZD, Scotland; 5 Military Nutrition Division, U.S. Army Research Institute of Environmental Medicine (USARIEM), Natick, MA 01760, USA; 6 College of Pharmacy and Nutrition, University of Saskatchewan, Saskatoon, SK S7N 5E5, Canada

Review articles are increasingly popular with authors, readers and journals, and the *British Journal of Nutrition* (*BJN*) now receives a large number of manuscripts for consideration each year. The aim of this Editorial, commissioned by the Editor-in-Chief of the *BJN*, is to help authors to determine the suitability of their proposed reviews for the journal, as well as their suitability for other Nutrition Society journals. We also provide guidance on what makes a successful submission.

The journals of the Nutrition Society offer a very wide range of review types. These extend from long, detailed articles in Nutrition Research Reviews, which often have a strong historical background, to the invited papers published in the Proceedings of the Nutrition Society, which describe contents of lectures from conferences on individual topics, often within a fixed scientific theme. Between these article types are up-to-date review articles published in the *BJN*, *Public Health Nutrition* and the *Journal of Nutritional Science* (*JNS*). The ‘Horizons Articles’ published in the *BJN* are shorter than Review articles and aim to critically evaluate recent novel developments that are likely to produce substantial advances in nutritional science. These articles should be thought-provoking, possibly controversial and be written by experts in the field. We look forward to receiving submissions which may be invited or submitted directly by interested authors.

The reviews in the *BJN* and *Public Health Nutrition* are given the prefix RE by the Editorial Office and include systematic reviews (prefix RS) and systematic reviews with meta-analysis (prefix RSMA) or HA for Horizons articles. Manuscripts that the *BJN* are unable to accept because of pressures on print space but are still of considerable merit may be offered the opportunity to be transferred to *JNS*. If this happens, the same referee and Editorial team handling the original *BJN* article will be retained, to help expedite the review process. Review articles may also be submitted to *JNS* directly.

To determine if the article is suitable for the *BJN*, authors may submit an abstract or précis of their article, prior to formal submission, to see if their article falls within the remit of the journal. Usually a reply will be given within a week or two, often with suggestions of how to strengthen the content to fit better within the *BJN* remit or to suggest a more appropriate journal, sometimes one of the other journals published by the Nutrition Society.

Some common reasons that we are unable to accept manuscripts for publication are given below. We hope this will be useful for those preparing manuscripts.

1. *Insufficient nutrition content*. Every journal has its own niche in the plethora of journals who seek the attention of authors and readers. For the *BJN*, it is made clear in the Instructions for Contributors that advancement of nutritional knowledge is the main criterion for acceptance and this also applies to submitted reviews. Nonetheless, we sometimes receive manuscripts that contain very little to no nutritional content. These often include very high-quality metabolic, biochemical, clinical or environmental review articles that lack sufficient nutrition content (dietary, food, nutrient or nutrient-derived metabolite concentrations) to be suitable for the *BJN*. The Discussion should therefore justify how the review advances nutritional knowledge. The Editorial Office is happy to offer advice on what is considered a nutrient.

2. *Scientific depth*. For readers, reviews in the scientific literature provide a good précis of a research field and they are often cited by authors in a global context in the Introduction and Discussion of original articles. For this reason, it is essential that review authors apply the highest rigour for their text and that it is checked by excellent critical peer review prior to publication. If not, problems can potentially arise by creating or maintaining scientific ‘myths’ or over-simplification. Among the manuscripts received, those that lack scientific depth often seem to be outside the area of expertise of the authors (e.g. as judged by a check of the article bibliography). Usually these articles are well-researched in terms of database searching but we find that the Discussion is may be at a superficial level and the reviews show a lack of the depth in understanding that would be expected from researchers who are active in the appropriate scientific field. To avoid these issues, we encourage authors to consult with a known international expert in the field and invite him/her to be a co-author in order to strengthen the Discussion (and perhaps elements of the Results).

3. *Language*. The Nutrition Society journals never reject a paper based on language problems, but on the other hand they are not always processed further (including external reviewing) until ambiguities or uncertainties as to meaning are corrected. We recognise that English is not the first language for many of our Reviewers and our Readership. Therefore, it is important that all manuscripts are written clearly and are easy to follow, so as not to cause confusion or uncertainty. If appropriate, the Editor will recommend that the manuscript would benefit from editing by someone well-versed in writing in scientific English. Sometimes authors use commercial companies to complete the editing process, for further details, see ‘Instructions for Contributors’. The important point is that whoever does the editing should have a reasonable scientific background and can therefore understand how to make the various points clear to the readership.

4. *Specific problems with Systematic Reviews without (RS) or with meta-analysis (RSMA)*. A systematic review or meta-analysis of randomised trials and other evaluation studies must be accompanied by a completed Preferred Reporting Items for Systematic Reviews and Meta-Analyses (PRISMA) Statement checklist, a guideline to help authors report a systematic review and meta-analysis^(1)^. Meta-analysis of observational studies must be accompanied by a completed Meta-analysis of Observational Studies in Epidemiology (MOOSE) reporting checklist, indicating the page where each item is included^(2)^. Manuscripts in these areas of review will be returned to the authors and not sent for peer review unless accompanied by the relevant completed checklist.

Meta-analyses are statistically complex, and the statistics are continually evolving. For statistical reasons, as well as other reasons mentioned in this Editorial, fewer than 20 % of RSMA submitted manuscripts were accepted in 2018. The availability of commercial software that computes meta-analysis results has weakened many RSMA manuscripts in instances where the authors have ignored the many constraints needed to ensure the statistics are conducted properly and the outcomes are fully understood. This is a specialised field of statistics, and even excellent statisticians can lack an understanding of the many subtleties and advances in approaches contained within this area. Authors are encouraged to consult a statistician who specialises in meta-analysis, either for advice or as a potential co-author.

The Introduction should clearly outline why the submitted manuscript is timely and necessary, with reference to relevant reviews previously published. Discussions in RS and RSMA articles should not be included in the Results. Discussion should identify the main outcomes and how this impacts nutrition. This should also be linked to the aims and objectives identified in the Introduction. Furthermore, to be published, RS or RSMA articles should add to the current knowledge base (or challenge existing knowledge) in a particular field of nutritional science, and this should be prominently mentioned in the text. Confirming previous RS or RSMA articles is not an acceptable conclusion. Either new knowledge (that adds to our understanding of the topic) needs to be included or, if the outcome(s) contradicts previous conclusions, then a strong explanation for why such differences occur needs to be discussed. This may relate to cohort type, populations studied, experimental design, over-reliance on a heavily weighted study, or simply previous inadequate number of studies or the number of individuals compared. Avoid non-specific terms such as ‘high’ or ‘moderate’ or ‘low’ intakes and provide actual ranges.

It would be more informative in the conclusions to avoid bland statements such as ‘more research is required in this area’. Instead, identify the key question that needs to be answered and provide details of the type of study that should be conducted, for example, ensuring a particular measure be included in future comparisons, standardisation of methods applied, etc.

5. *Up-to-date nature of the article*. The *BJN* expects all review articles to be up-to-date. As an approximate guide, database searches for RS and RSMA should be completed within 3 months of the initial submission date to the *BJN*. Deputy Editors will return the manuscript to authors at the pre-screening phase with a mandatory request for major revisions if the article is not up-to-date. This is important since more recent searches may alter the conclusions (RS) or the statistical analysis (RSMA).

Searches should be from at least three separate databases. Authors should avoid those that are already nested within other databases, as occurs with the PubMed family. Using more than three databases is encouraged. The date and month of the last database search should be stated in the article and search terms should be clearly identified – either directly in Materials and Method or in a text Table or Appendix Table.

6. *RSMA*. Among the many problems specifically encountered with RSMA are: not understanding when random or fixed effects models should be applied, not describing which of the several fixed or random effects models are selected (and why), combining cohorts of different robustness in a single analysis (e.g. randomised controlled trials and case–control studies) or combining different outputs in the same analysis (e.g. blood pressure and a plasma hormone or metabolite). Furthermore, often use is made of comparison of highest and lowest tertiles from the searched publications. However, the highest tertile in one study may be less than the lowest in another (and vice versa) and so the outcome does not help nutritionists understand what is an optimal intake of a food or nutrient. This is particularly so when near-pharmacological doses may be used at the highest tertile, or severe deficiency represents the lowest tertile. When possible, a dose–response analysis should be attempted, and with sufficiently robust data, this should provide values of use to our readership, showing where intake responses plateau, and assist in providing recommendations for individuals or populations.

Further discussion of the many problems of RSMA and how to best process data in nutritional science are contained in a recent *BJN* article^(3)^. This is required reading for any authors wishing to submit an RSMA.

7. *Retracted articles*. A number of published papers used in a review (or even research) article may be retracted between initial submission and final acceptance. It is important to check for retracted articles at each stage (revisions) of the referee process because their inclusion may impact on conclusions or where statistical analysis within an RSMA has been conducted. The Web of Science database now indexes these types of studies with the term ‘retracted’. Other databases may (or will in the future) include this facility.

8. *Summary*. Attention to the Instructions for Contributors on the rationale of advancing nutritional knowledge will help focus the Introduction (including aims and hypotheses) and this will help in presentation of the Discussion. The majority of our Readership are nutritionists or nutrition scientists, and they may be looking for information presented in a way to help them advise individuals, populations, advisory bodies or governments on the correct nutrient requirements. We recommend that authors who are inexperienced or even new to a research area should not hesitate to seek advice (perhaps as collaboration) from leaders in the field. For RSMA articles, seek the best statistical advice possible. Avoid simple comparison of highest (which may be near pharmacological, or at least out of the range of normal possible intakes) and lowest (which may involve populations in severe deficiency conditions) tertiles. Dose–response analysis is encouraged.

The *BJN* prides itself on the rigour applied to refereeing review articles which ultimately benefits our authors and readership.

## Disclaimer

The opinions or assertions contained therein are the private views of the authors and are not to be construed as official views of the authors' host institutions, including reflecting the views of the US Army or the US Department of Defense. Any citation of commercial organisations and trade names do not constitute an official US Department of the Army endorsement of approval of the products or services of these organisations.
